# Epidemiology of 2017 influenza outbreaks in nine Australian Aged care facilities

**DOI:** 10.1111/irv.12811

**Published:** 2020-10-07

**Authors:** Elmira Hooshmand, Aye Moa, Mallory Trent, Mohana Kunasekaran, Christopher J. Poulos, Abrar Ahmad Chughtai, C. Raina MacIntyre

**Affiliations:** ^1^ Biosecurity Program Kirby Institute University of New South Wales Sydney NSW Australia; ^2^ Aged Care Clinical Services HammondCare Sydney NSW Australia; ^3^ School of Public Health and Community Medicine University of New South Wales Sydney NSW Australia

**Keywords:** 2017 season, aged care, Australia, influenza, influenza outbreak

## Abstract

**Background:**

The 2017 A/H3N2 influenza season was the most severe season since the 2009 influenza pandemic. There were over 591 influenza outbreaks in institutions across the state of New South Wales (NSW) in Australia.

**Aim:**

To describe the epidemiology of influenza outbreaks in nine Sydney aged care facilities in 2017.

**Methods:**

Study data were collected from nine Sydney aged care facilities for 2017 influenza season. Descriptive epidemiological analysis was conducted.

**Results:**

From the nine sites included, with a total of 716 residents, four sites reported laboratory‐confirmed influenza outbreaks during the study period, with an attack rate in residents ranging from 6% to 29%. The outbreaks resulted in lockdowns in two facilities and hospitalisation of seven residents. No deaths were reported as a result of influenza infection. Influenza A was the most common influenza type reported across the facilities. The duration of outbreak lasted for 1‐4 weeks varied by site.

**Conclusion:**

The 2017 season was a severe influenza season recorded in Australia. About half of the facilities studied experienced outbreaks of influenza, with a high attack rate among residents. Infection prevention and control measures and outbreak management plans are crucial for aged care facilities, including vaccination of staff and visitors to prevent outbreaks among the vulnerable residents.

## 1 BACKGROUND

Influenza is a highly infectious respiratory illness that causes seasonal epidemics.^1^ Globally, influenza impacts 3‐5 million people per year, resulting in over 650 000 deaths.^2^ In Australia, influenza infections result in approximately 18 000 hospitalisations a year, primarily affecting young children, older adults, and those with immunocompromising conditions.^2^, ^3^, ^4^

Residents in aged care facilities (ACF) are at high risk of influenza infections and respiratory complications.^5^, ^6^ Advanced age, frailty and comorbidities can result in reduced immune function.^7^ Common clinical signs and symptoms of influenza include fever, cough, sore throat, nasal congestion, muscle aches and pain, and fatigue.^8^ However, among elderly populations, the person can be afebrile or may have atypical symptoms.^9^ As such, a clinical diagnosis can be missed by nurses and aged care staff. Use of antiviral drugs such as oseltamivir, which if taken within 48 hours after the initial symptoms, can reduce disease severity and influenza‐associated complications,^10^ and studies have shown impact of using oseltamivir as prophylaxis in preventing and management of influenza in the residential care facilities.^11^, ^12^, ^13^ One study reported that oseltamivir prophylaxis reduces the influenza attack rate of 90% in residents during outbreaks, particularly in high care wards.^11^ Furthermore, crowded living conditions and the frequent use of common rooms favour a rapid spread of respiratory infections. If health and safety precautions are not practised adequately, this can result in high infection transmission,^5^, ^6^ and as a result, frequent outbreaks of influenza infection can occur in residents in ACF. The introduction of influenza to aged care settings may come from staff working in the aged care home and/or the visitors to the site.^14^ In ACF, staff members have frequent contact with the residents, and thus, there is a high risk of transmission to residents.^15^

In Australia, the 2017 influenza season had the highest number of laboratory‐confirmed influenza notifications, at a rate of 1021.6 per 100 000 population,^16^, ^17^ and resulted in 29 000 hospital admissions and 745 deaths.^16^ However, the severity of infection varies with the type of seasonal strain circulating during the season. In 2017, influenza A/H3N2 was the predominant strain in circulation. There was a total of 591 confirmed influenza outbreaks in institutions reported in New South Wales (NSW) alone.^18^ Additionally, the influenza vaccine has a lower vaccine effectiveness in older adults^16^, ^19^ compared to other age groups in general. To measure the effect of influenza, in this study, we aimed to estimate the impact of the 2017 influenza season in nine Australian aged care homes.

## 2 METHODS

The study was conducted in nine ACF in affiliation with a multisite aged care provider in Sydney. The inclusion of study sites was chosen by all facilities located in metropolitan Sydney. Facilities outside Sydney and in other States were not included. ACF managers were interviewed and managers completed the study questionnaires retrospectively in 2018 about 2017 influenza outbreaks. An outbreak was defined in the study as two or more cases of laboratory‐confirmed influenza occurring concurrently within the same facility in a week. Nasopharyngeal swabs were collected for symptomatic residents, and diagnosis of influenza was confirmed by real‐time reverse transcription‐polymerase chain reaction.

Each site was categorised as dementia‐specific low care facilities, regular ACF and high care with advanced dementia ACF. High care facilities were for residents who were considered immobile. For this study, immobile was defined as the resident’s inability to perform tasks independently. Low care facilities were for residents who were mobile and might require less assistance with their daily routines.

For the study, de‐identified aggregated data were collected for residents and staff from each ACF; thus, individual data were not available. Baseline data and outbreaks information were entered from nine participating sites. Descriptive analysis of influenza outbreaks in 2017 was conducted. Outbreaks were analysed by predictors such as demographics of residents, number of rooms and shared rooms in each facility, the number of outbreaks, outbreak duration, lockdowns during the outbreak, total number of laboratory‐confirmed cases, total number of influenza‐related hospitalisations and deaths in each facility. We also documented start and end date of outbreak, speculated origin of transmission, vaccination of staff and residents, number of staff and workplace vaccination policy for staff (vaccine service and reimbursement). Staff data were incomplete; thus, data analysis was focused mainly on residents in the study. Attack rate of influenza was calculated for residents using the number of laboratory‐confirmed cases of influenza infection and the total number of residents (potentially who were exposed) in the facility.

Ethics approval was granted from UNSW HREC and the research and governance office of ACF (Approval number: HC17996).

## 3 RESULTS

There were nine facilities in affiliation with an Australian aged care provider included in the study. Each site was independently run with separate staff and managing team. Each site had common kitchen and dining areas where residents can interact daily, and all facilities had a basin and handwashing facilities in common areas and most private rooms. Table 1 described the demographics of residents by the facility. There was a total of 716 residents reported across nine aged care sites. Age of residents ranged from 57 to 101 years, and there were a high number of female residents in the facility (Table 1).

**Table 1:** Demographic characteristics of residents in aged care facilities

Aged care facility & type of aged care provided	Age of residents (range, y)	Number of male residents, n (%)	Number of female residents, n (%)	Total number of residents (N)	Total number of beds/Number of rooms with single bed
Facility A (Dementia‐specific, low care)	62‐96	13 (23%)	44 (77%)	57	40/40
Facility B (Aged care)	73‐101	7 (11%)	55 (89%)	62	62/62
Facility C (Aged care)	70‐99	13 (54%)	11 (46%)	24	24/24
Facility D (Dementia‐specific, Residential/Aged care)	65‐92	36 (48%)	39 (52%)	75	57/57
Facility E (High care/ Aged care)	64‐95	8 (13%)	52 (87%)	60	60/28
Facility F (Dementia‐specific)	77‐95	14 (37%)	24 (63%)	38	40/40
Facility G (High care & Low care)	61‐97	49 (26%)	140 (74%)	189	124/117
Facility H (Residential care)	65‐99	Unavailable (‐)	Unavailable (‐)	128	92/92
Facility I (Dementia‐specific, high care)	57‐101	38 (46%)	45 (54%)	83	83/83
Total	57‐101	178^a^	410^a^	716	‐

^a^ Data not included for facility H.

Only 2/9 facilities (E and G) had shared rooms with more than one bed. From 124 beds, Facility G reported seven shared rooms with an average of two beds per room. Facility E had 12 shared rooms, with an average of two beds per room.

Table 2 shows information on vaccination availability for staff by sites. A total number of 689 staff were recorded in all facilities. Facilities D and H were the only facilities that provided onsite vaccination for staff members. It was also reported that influenza vaccination for staff was mostly carried out by the private healthcare providers. However, information was not available for the number of staff who received influenza vaccination in 2017.

**Table 2:** The availability of seasonal influenza vaccine for staff by facilities^a^

Aged care facility	Number of staff	Is influenza vaccination routinely reimbursed for staff?	Vaccination site
Facility A	38	Yes	Private GP
Facility B	79	Yes	Private GP
Facility C	Unavailable	Unknown	Private GP
Facility D	70	Yes	Both through facility and private GP
Facility E	108	Yes	Private GP
Facility F	31	Yes	Private GP
Facility G	125	Yes	Private GP
Facility H	115	Yes	Both through facility and private GP
Facility I	123	Yes	Private GP

^a^ Data not included for facility C.

Table 3 summarises data on facilities that reported influenza outbreaks involving residents and/or staff during 2017 influenza seasons. Of all nine ACF in the study, facility D, F, G, H and I reported a total of 41 laboratory‐confirmed cases of influenza among residents in 2017, with attack rates in residents ranged from 6% to 29%. The other four facilities reported no cases of confirmed influenza infection. Facility D reported two confirmed cases of influenza with unknown viral strains in the study. However, facility D did not meet outbreaks criteria set out for the purpose of this study; thus, the information from facility D was not included in Table 3. Facility G reported the cause of initial viral transmission to be from a resident. However, facility H reported initial viral transmission from a staff member. The three facilities reported that outbreaks lasted for duration of 1‐4 weeks, and two facilities (facility F and G) reported lockdown as a result of the outbreak in the facility (Table 3). Data on vaccination status of residents with seasonal influenza vaccine were not available in the study.

**Table 3:** Reported influenza outbreaks in aged care facilities in 2017

	Facility F^a^	Facility G	Facility H	Facility I
First case of influenza in facility	Unknown	17/07/2017	14/08/2017	28/07/2017
Speculated origin of transmission	Unknown	Resident	Staff	Unknown
Outbreak (start date)	Unknown	19/07/2017	8/10/2017	31/07/2017
Outbreak (end date)	Unknown	7/08/2017	28/10/2017	8/08/2017
Duration of outbreak	Unknown	3 wk	4 wk	1 wk
Number of times facility was locked down due to outbreak	2	1	0	0
Number of residents in facility during outbreak	38	124	92	83
Number of laboratory‐confirmed influenza cases among all residents (attack rate, %)	11 (29%)	17 (14%)	6 (6.5%)	5 (6.0%)
Influenza virus	Influenza A/Influenza B	Influenza A	Influenza A	Influenza A
Number of influenza‐related hospitalisations in residents	1	4	0	0
Number of influenza‐related deaths in residents	0	0	0	0
Number of staff in facility during an outbreak	Unknown	Unknown	131	55
Number of laboratory‐confirmed influenza cases among staff	Unknown	2	4	Unknown
Number of influenza‐related hospitalisations in staff	Unknown	0	0	0

Note

No outbreak reported from facility D, thus not included in the table.

^a^ Facility F did not provide data on start and end date of outbreaks, the number of residents and staff affected during each outbreak, and speculated origin of transmission.

Influenza type A was the most common subtype across all facilities. Facility G had the highest number of influenza infection (n = 17) among residents; mostly were influenza type A viruses. It was followed by facility F and H, with the total number of cases (n = 11 and n = 6), respectively. The facility I reported a total of five confirmed cases during that time. Among the total, six confirmed cases of influenza B infection were reported by facility F. No other sites reported cases of influenza B in the season. Three facilities, facility D, F and G, reported influenza‐related hospitalisations among the residents (a total of 7 cases; 2, 1 and 4 cases respectively). No deaths were reported due to influenza‐related illnesses in these outbreaks.

## 4 DISCUSSION

Outbreaks of influenza in aged care settings are well recognised. We showed a high rate of influenza outbreaks in ACFs in 2017. Our study recorded the influenza attack rate of 6%‐29% in residents across the facilities. Consistent to our finding, a literature review of infectious diseases outbreaks reported that the median attack rate of influenza outbreaks in residents was 33% (ranged 4%‐94%) in the elderly in long‐term care facilities.^20^ In 2017, there were 591 institutional outbreaks of influenza in NSW alone.^15^ It resulted in a high healthcare burden nationally, which had a significant economic impact on the Australian healthcare system.^16^ A high rate of influenza in the community with increased influenza‐related hospitalisations and deaths as well as numerous, severe ACFs outbreaks were reported in 2017 across the country.^21^, ^22^, ^23^, ^24^ In that year, influenza A/H3N2 circulated, and the overall vaccine effectiveness of −3% and −20% for A/H3 was reported in adults ≥65 years in Australia, resulted in a high disease burden in the elderly.^19^

From the study, four of the nine facilities reported influenza outbreaks in 2017. Studies have shown that highly populated residential homes have a higher risk of viral transmission, due to complexity in maintaining infection control practices in the facilities.^25^, ^26^ Moreover, many studies reported that transmission from staff members was the most common spread of infection in aged care homes, who then transmit to residents.^27^, ^28^, ^29^ However, in this study, the speculated origin of transmission was reported from both staff and residents in the ACFs. This may have occurred as a result of third party transmission between residents and visiting family members.^28^, ^29^ High vaccination rates in staff and family members who regularly visit the sites are crucial in reducing influenza outbreaks in the aged care homes.

With regard to vaccination, low influenza vaccine uptake was reported among aged care staff.^30^, ^31^ Poor vaccination uptake among staff may have been due to several factors such as out of pocket expenses for yearly vaccination, inconvenience of vaccination site for staff, busy working schedules, varied vaccination requirement by sites and individual attitudes towards vaccination.^32^ In our study, we found that two out of nine facilities offered onsite vaccination for staff members and the remaining seven sites offered reimbursements for staff once the vaccination was done. However, the provider only reimbursed the cost of the vaccine and none for any associated GP consultation fees. We previously found that higher vaccine uptake was observed for staff in facilities where vaccination is offered onsite.^30^ In addition, in facilities where staff who come from low socio‐economic backgrounds, low vaccination uptake may be due to initial out of the pocket expenses for staff such as the cost of seeing a private GP, which can be a further barrier in receiving the vaccination.^14^, ^30^ resulting in less voluntary uptake of vaccination among staff in facilities that do not offer onsite vaccination. Due to numerous outbreaks of influenza in aged facilities in 2017, the Australian Aged Care Quality Agency took a national survey to review the uptake of seasonal influenza vaccination among staff.^33^, ^34^ After the review, in subsequent influenza seasons, Australia’s health policymakers implemented a compulsory vaccination of aged care staff to achieve the targeted vaccination uptake nationally.^33^, ^35^

Numerous studies have shown that the spread of influenza is more rapid in facilities where shared rooms are used.^36^, ^37^, ^38^ This is due to the likelihood of contact transmission through close living arrangements and sharing of utilities and common areas among the residents.^25^, ^26^, ^37^ Residents sharing a room with an influenza‐infected roommate are about three times more likely to develop symptoms than residents in single rooms.^39^ Studies have shown that strict hygiene practices such as the use of PPE among staff can significantly reduce transmissibility of influenza.^40^, ^41^ Control of the spread of infection can be challenging to manage in facilities with a higher number of residents.

This study has limitations. First, it only provides a cross‐sectional picture of the reported influenza outbreaks among the participating ACFs in 2017, where a higher number of influenza A/H3 virus circulated, resulting in a severe influenza season across the nation. Second, our findings may be underestimated as residents may have been missed due to mild or asymptomatic cases during these outbreaks. Third, we did not have adequate data on staff, thus limiting information and understanding about the staff working in ACFs in the study.

Despite the high vaccination coverage among residents, older adults had been hit hard by influenza infection. About 85% of those that were vaccinated in 2017 and exposed to the virus were affected by influenza in that year.^42^, ^43^ In Australia, the 2017 seasonal influenza vaccine was reported to have reduced vaccine effectiveness of 33% overall and only 10% effectiveness against A/H3N2 strain.^19^ Additional reasons for low vaccine effectiveness may have been due to genetic diversity in circulating A/H3N2 and the vaccine strain, as well as waning protection against influenza virus especially in older adults following vaccination.^44^ For control of influenza outbreaks in the ACFs, high vaccine coverage of staff and residents, training of staff on infection control measures and the use of effective surveillance system are essential to monitor and prevent such outbreaks in these settings.^40^, ^45^, ^46^

## CONFLICT OF INTEREST

CR MacIntyre has received funding for investigator‐driven research separate from this study, from Sanofi and Seqirus, and has been on advisory boards for the same for the last 5 years. Other authors have none to declare.

## AUTHOR CONTRIBUTION

**Elmira Hooshmand:** Data curation (equal); Formal analysis (equal); Writing‐original draft (lead); Writing‐review & editing (equal). **Aye Moa:** Formal analysis (equal); Supervision (supporting); Writing‐review & editing (equal). **Mallory Trent:** Supervision (supporting); Writing‐original draft (supporting); Writing‐review & editing (equal). **Mohana Kunasekaran:** Data curation (equal); Project administration (lead); Writing‐review & editing (supporting). **Christopher Poulos:** Conceptualization (supporting); Supervision (supporting); Writing‐review & editing (equal). **Abrar Chughtai:** Supervision (supporting); Writing‐review & editing (equal). **C. Raina MacIntyre:** Conceptualization (lead); Supervision (lead); Writing‐review & editing (equal).
